# The Physical and Genetic Framework of the Maize B73 Genome

**DOI:** 10.1371/journal.pgen.1000715

**Published:** 2009-11-20

**Authors:** Fusheng Wei, Jianwei Zhang, Shiguo Zhou, Ruifeng He, Mary Schaeffer, Kristi Collura, David Kudrna, Ben P. Faga, Marina Wissotski, Wolfgang Golser, Susan M. Rock, Tina A. Graves, Robert S. Fulton, Ed Coe, Patrick S. Schnable, David C. Schwartz, Doreen Ware, Sandra W. Clifton, Richard K. Wilson, Rod A. Wing

**Affiliations:** 1 Arizona Genomics Institute, Departments of Plant Sciences and Ecology and Evolutionary Biology, BIO5 Institute for Collaborative Research, University of Arizona, Tucson, Arizona, United States of America; 2 Laboratory for Molecular and Computational Genomics, Department of Chemistry, Laboratory of Genetics, University of Wisconsin-Madison, Madison, Wisconsin, United States of America; 3 Plant Genetics Research Unit, USDA-ARS, and Division of Plant Sciences, University of Missouri, Columbia, Missouri, United States of America; 4 Cold Spring Harbor Laboratory, Cold Spring Harbor, New York, United States of America; 5 The Genome Center at Washington University School of Medicine, St. Louis, Missouri, United States of America; 6 Center for Plant Genomics, Iowa State University, Ames, Iowa, United States of America; 7 Department of Genetics, Washington University School of Medicine, St Louis, Missouri, United States of America; The Salk Institute for Biological Studies, United States of America

## Abstract

Maize is a major cereal crop and an important model system for basic biological research. Knowledge gained from maize research can also be used to genetically improve its grass relatives such as sorghum, wheat, and rice. The primary objective of the Maize Genome Sequencing Consortium (MGSC) was to generate a reference genome sequence that was integrated with both the physical and genetic maps. Using a previously published integrated genetic and physical map, combined with in-coming maize genomic sequence, new sequence-based genetic markers, and an optical map, we dynamically picked a minimum tiling path (MTP) of 16,910 bacterial artificial chromosome (BAC) and fosmid clones that were used by the MGSC to sequence the maize genome. The final MTP resulted in a significantly improved physical map that reduced the number of contigs from 721 to 435, incorporated a total of 8,315 mapped markers, and ordered and oriented the majority of FPC contigs. The new integrated physical and genetic map covered 2,120 Mb (93%) of the 2,300-Mb genome, of which 405 contigs were anchored to the genetic map, totaling 2,103.4 Mb (99.2% of the 2,120 Mb physical map). More importantly, 336 contigs, comprising 94.0% of the physical map (∼1,993 Mb), were ordered and oriented. Finally we used all available physical, sequence, genetic, and optical data to generate a golden path (AGP) of chromosome-based pseudomolecules, herein referred to as the B73 Reference Genome Sequence version 1 (B73 RefGen_v1).

## Author Summary

Maize has been a cultural icon and staple food crop of Americans since the discovery of the new world in 1492. Contemporary society is now faced with growing demands for food and fuel in the face of global climate change and the potential for increased disease pressure. To provide a comprehensive foundation to systematically understand maize biology with the goal of breeding higher yielding, disease-resistant, and drought-tolerant cultivars, our consortium sequenced the B73 genome of maize. In this study, we used a comprehensive physical and genetic framework map to develop a minimum tiling path (MTP) of over 16,000 BAC clones across the genome. The MTP was generated dynamically and integrated numerous data types, such as in-coming genome sequence, over 8,000 sequence-based genetic markers, and the maize optical map. This allowed us to genetically anchor, order, and orient the majority of the maize physical map and genome sequence to the genetic map. Post-genome sequencing, we constructed a golden path (AGP) of sequence-based pseudomolecules representing the ten chromosomes of the maize B73 genome (B73 RefGen_v1). This unprecedented integration of genetic, physical, and genomic sequence into one framework will greatly facilitate all aspects of plant biological research.

## Introduction

Maize is an important crop and a model biological system. With global climate change and increasing caloric and raw material demands, the development of higher yielding and more stress-resistant maize cultivars is a major challenge facing 21^st^ century breeders. Approximately 50 million years ago maize shared a common lineage with all grass and cereal ancestors [1]. Subsequently, the maize ancestor underwent allotetraploidization and diploidization [2]–[5], prior to domestication some 10,000 years ago in the Americas. The present day maize genome is genetically diploid (n = 10), and has a genome size (GS) of approximately 2300–2700 Mb [6], 85% of which is composed of transposable elements [7]. With the smaller and less complex cereal genome sequences of rice (GS = 389 Mb; [8]) and sorghum (GS = 700 Mb; [9]) already completed, the generation of a whole genome sequence of maize offers the greatest technical challenge to date for any complex plant genome.

Since 1998 the U.S.A. National Science Foundation's Plant Genome Research Program has invested heavily in the development of resources and pilot projects to build a foundation to sequence the maize genome, including generation of maize genetic [10]–[14], physical [15]–[18], and optical maps [19], sequencing maize gene space by methylation filtration and high *C_o_t* selection [20]–[22], BAC end sequencing [23], random BAC sequencing [24], sequencing large contiguous maize regions [25], and the maize full-length cDNA project [26]. These investments came to fruition in 2005 with the funding of the Maize Genome Sequencing Consortium (MGSC) to use a novel clone-by-clone approach to sequence the genome of the maize inbred B73, a process that was completed in 2009 [7].

Here we present a detailed account of the utilization of a previously described genetically-integrated sequence-ready physical framework map of the B73 maize genome (721 contigs anchored with 1092 genetic markers, covering ∼94% of the genome [18]) as the *vade mecum* to dynamically select a minimum tiling path (MTP) of BAC clones across the genome. We describe our progress in integrating new and more complex resources into the physical map to better guide the generation, validation and annotation of a reference genome sequence for maize. These processes included the use of maize genome sequence and optical map information to merge, break, anchor and orient FPC contigs. Upon completion of the shotgun sequencing and sequence improvement of most large-insert clones, we combined all available evidence (i.e. sequence, physical, genetic, and optical map information) to construct a golden path (AGP) of pseudomolecules across the maize genome, hereinafter referred to as the “B73 RefGen_v1”.

## Results/Discussion

### Generation of a Minimum Tiling Path (MTP) of Bacterial Artificial Chromosome (BAC) and fosmid clones to sequence the B73 maize genome

To sequence the maize genome (B73), we employed a clone-by-clone approach and selected a minimum tiling path of BACs across the integrated genetic and physical map. Initially we selected 3,200 BACs that were spaced approximately 800 kb apart across the genome. Additional criteria used to select these “seed” BAC clones were: 1) each had a genomic insert that was larger than the average insert sizes of the BAC libraries; 2) each had a pair of high-quality end sequences; 3) each had a high-quality fingerprint; and 4) where possible, each had an associated genetic and/or overgo marker [27]. These combined criteria ensured that the genomic position of each seed BAC clone was known, that each clone could be easily validated prior to shotgun library construction/sequencing, and that a maximum amount of sequence could be obtained from each region due to the large clone insert size.

Because the previously published B73 maize BES data set [23] was not adequate to walk from seed BACs, the MGSC resequenced BAC ends for the ZMMBBc *Eco*R1/*Mbo*I BAC library, resulting in a total of 340,869 new BESs to aid clone walking/sequencing. The ZMMBBc library was selected because it had the larger average insert size of the two BAC libraries used to generate the physical map. Combined, we employed 815,473 BESs (70% paired) for the maize genome sequencing project. In addition, the MGSC also generated a total of 827,571 (72% paired end) fosmid end sequences/trace files that were used primarily for MTP gap filling (see below).

**Figure 1:**
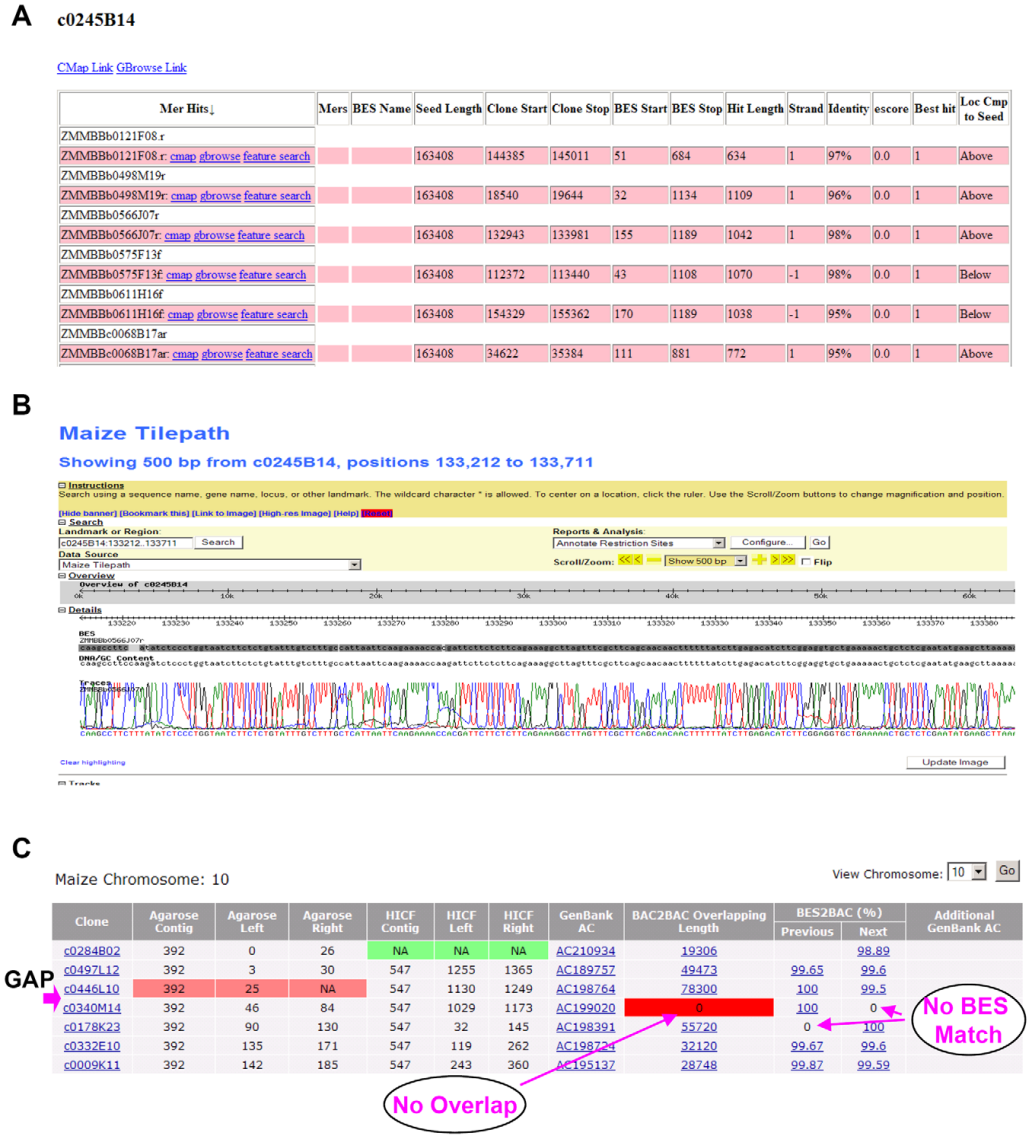
Sequencing pipeline for MTP clone selection and gap analysis. (A) An example of STC-based clone walking. Candidate walking clone list for seed BAC c0245B14. The list showed clones in which BES shared >95% sequence identity with the seed BAC; (B) Gbrowse view of sequence and trace alignment of candidate clone b0566J07 to seed BAC c0245B14. (C) Gap analysis pipeline to check gaps between adjoining clones.

Once a seed BAC was sequenced we employed one of two methods to select adjacent BAC clones that had minimal sequence overlap. The first method, termed the sequenced tagged connector (STC) approach [28], utilized the BES data set (FASTA and trace files) to identify BESs that minimally aligned to the seed BAC sequence on either side of the sequence. Once a MTP BAC clone was identified, its position on the physical map was checked, then validated by BAC end sequencing prior to incorporation in the production sequencing pipeline. To make MTP clone selection more efficient, we developed a web-based MTP Tilepath pipeline interface (Figure 1A and 1B) that is described in detail in Text S1.

The second method used for MTP selection relied solely on the underlying BAC fingerprints used to assemble the maize integrated genetic and physical map. This method was employed due to the scale of the project and the timeline mandated to complete the project. It simply was impossible to exclusively use the STC approach, because the improved seed-BAC and MTP-walk sequences were not generated rapidly enough to supply the shotgun library and production sequencing pipelines with adequate numbers of BACs to complete the project on time. To select MTP BAC clones for sequencing with fingerprints, we used an e-value score of e^−9^ to e^−15^ between adjacent BAC clones in the maize high information content fingerprint (HICF) map [17] to ensure minimal overlap. E-value scores for evaluating fingerprint overlap were assessed using the FPC Analysis function [29] and resulted in an average overlap of adjacent BAC clones of 38 kb across the genome. Such overlap can thereby exclude false overlaps created by two identical (or nearly identical) retrotransposons whose sizes are normally less than 15 kb. This e-value parameter could be used for MTP selection of other genomes where HICF physical maps are available.

The final step in MTP generation was to check and fill gaps with either BAC or fosmid clones. To simplify this task, we developed a comprehensive web-based MTP interface (Figure 1C) that is described in Text S1. To ensure high-confidence overlap between two contigs we set the following criteria: 1) two adjoining clones must have overlap in Megablast searches with over 99.9% sequence identity; 2) the highest scoring overlap must be between each clone, and not with any other clone in other parts of the genome; 3) the BES of one clone must align to the sequence of its adjacent clone with over 95% identity; and 4) if the sequence identity in the BAC-end search was less than 99%, the sequence alignment along with the trace chromatograph was manually checked. If any one of these criteria was not met, the clone was flagged and manually annotated.

In conclusion, we selected a total of 16,910 MTP clones across the maize genome (3,200 seed, 5,748 STC walks, 6,048 FP walks, 1,795 BAC gaps and 63 Fosmid gaps, and 56 BACs from outside projects). The full list of MTP clones and an interactive website can be accessed at http://www2.genome.arizona.edu/genomes/maize and in Table S1.

### Improvement of the maize integrated genetic and physical map

In our previous study [18], we were unable to merge or genetically anchor additional FPC contigs based on fingerprint evidence alone. By utilizing maize genome sequence and genetic map information we were able to significantly improve the physical map by performing new contig merges, breaking mis-assembled contigs and anchoring additional FPC contigs to the maize genetic map.

Using the same rules described above for gap checking, in combination with the maize genome sequence, we were able to perform 109 FPC contig merges, and identified ten FPC contigs that were incorrectly merged (Table S2). These latter contigs were broken apart and then merged into 17 new FPC contigs. This analysis resulted in a total of 435 FPC contigs in the maize physical map, which covered ∼93% (2120 Mb) of the 2300-Mb genome. In addition, 170 small low-coverage FPC contigs (∼25 Mb in total) shown to represent contaminating cotton sequences were removed from the physical map assembly. The contamination was identified by Kmer [30] and BAC end sequence analyses. All contaminated clones were from the ZMMBBb library and most likely originated during the BAC library construction process.

To fully integrate the physical map with the maize genetic map we utilized all publicly available marker data from the IBM2 2008 Neighbors Map (Schaeffer, Sanchez-Villeda, and Coe, 2008; http://maizegdb.org/map.php), and the literature. The IBM2 2008 Neighbors map contains 15,932 markers (11,475 publicly available). However, due to the long history of these genetic markers, dating back 20 years or more, the nucleotide sequences of many markers were not deposited into centralized databases, such as maizeGDB or GenBank. To integrate additional genetic markers at the sequence level, we conducted extensive literature and Google searches and identified 2,864 markers with sequences not associated with markers in maizeGDB or linked to GenBank entries.

In total, we obtained 9,229 sequence-based genetic markers with available sequences (http://www2.genome.arizona.edu/genomes/maize). Of these, 8,315 markers could be mapped onto both the physical map and the B73 RefGen_v1 (Table S3). We could not pinpoint the genomic locations of 134 markers (indicated as “no hit” in Table S3), perhaps due to lack of sequence or genome coverage in the related regions, or their origin as inbred-specific sequences. Gore et al. [31] reported that about 7.8% of the maize sequences could be inbred specific. The low genetic map resolution of these markers made it impossible to determine the cause for no coverage. Additionally, 780 markers were placed on different chromosomes in contrast to their reported genetic positions (Table S3). Most of these 780 markers were from low-resolution maps and their genetic positions could not be validated. Of the 90 bin markers (Table 1, partial; the full list is in Table S4) used to divide the maize genome genetically, we could confidently place 87 markers on both the physical map and the B73 RefGen_v1. There were three bin markers (RFLP markers umc5a, agrr37b, and csu93b) with physical positions that conflicted with their genetic positions. Most likely, those multiple copy markers were InDels that were present in different parental lines, but absent in B73 or in gaps, because each marker only had one locus in the B73 genome (RefGen_v1), instead of multiple ones in their original mapping parents.

**Table 1:** Position of bin markers in the B73 physical map and RefGen_v1^a^.

Marker	Chr	Bin	Genetic^b^	Original Map	Type^c^	Seq. Source^d^	Start^e^	End^e^	clone	FPC Ctg
tub1	1	1.01	2.5	IBM2	F	X52878	2022607	2024984	c0363D20	1
umc157a(chn)	1	1.02	114.4	IBM2	P	G10823	12357364	12357663	c0140E02	5
umc76a	1	1.03	198.4	IBM2	F	G10866	29364559	29364266	c0380M20	9
asg45(ptk)	1	1.04	294.3	Gnp2004	P	AY771210	52239800	52240131	b0109M14	12
csu3	1	1.05	405	IBM2	F	DQ123891	81360132	81360551	c0122B13	20
umc67a	1	1.06	496.6	IBM2	P	G13173	175505327	175505029	c0152A14	36
asg62	1	1.07	607.3	IBM2	F	DQ001865	198707401	198707865	c0479A09	41
umc128a	1	1.08	722.4	IBM2	F	umc128	227601774	227602233	b0310F15	46
cdj2	1	1.09	812.3	IBM2	F	AY109456	252192856	252193562	b0611E16	52
umc107a(croc)	1	1.1	886.9	IBM2	P	G10803	266927146	266927488	c0293G16	56
umc161a	1	1.11	963.6	IBM2	F	AY771212	282140672	282141394	c0086K08	61
bnl6.32	1	1.12	1113	IBM2	F	bnl6.32	296840063	296840574	c0455B14	63
bnl8.45a	2	2.01	3.3	Gnp2004	P	G10776	1546872	1547084	b0252P05	68
lox6	2	2.02	50.9	IBM2	F	AY771214	4175012	4174428	c0468P22	69
umc6a	2	2.03	164.8	IBM2	F	G10856	14920255	14920433	c0530G21	72
umc34	2	2.04	243.3	IBM2	F	DQ001866	28063927	28064503	c0030B11	74
umc131	2	2.05	342.4	IBM2	F	umc131	71031565	71031939	c0244C01	82
umc255a	2	2.06	364.5	IBM2	P	umc255	149697523	149697768	b0120F07	90
umc5a	7	2.07	405.8	Gnp2004	P	umc5	116650892	116650654	b0022A14	315
asg20	2	2.08	478.7	IBM2	F	DQ123894	201356132	201355954	c0158O02	103
umc49a	2	2.09	591.5	IBM2	F	DQ123895	219604574	219604915	c0184K09	108
php20581b(tb)	2	2.1	692.7	Gnp2004	P	G10795	231788583	231788397	b0109B01	109
umc32a	3	3.01	11.3	UMC98	P	umc32	1726276	1725856	c0286H14	111
csu32a	3	3.02	60	IBM2	F	DQ123896	3837012	3837402	c0299P11	111
asg24a(gts)	3	3.03	109	IBM2	P	AY771217	8405715	8482306	b0166B24	112
asg48a	3	3.04	152.7	IBM2	F	G13184	12862813	12862593	c0385I07	113
umc102a	3	3.05	297.9	IBM2	F	DQ005498	122406867	122407553	c0072M24	124
im30p1	3	3.06	391.4	IBM2	P	G10766	166733121	166732779	b0583P10	131
bnl6.16a	3	3.07	520.7	IBM2	F	G10768	189303505	189303133	c0328L01	138
umc17a	3	3.08	585.5	IBM2	F	AY771218	203506017	203506852	b0460H12	145
umc63a	3	3.09	697.2	IBM2	F	G10857	214210836	214210676	b0347M11	147
cyp1	3	3.1	845.2	Gnp2004	P	DQ005499	230486027	230486291	b0147G12	153

a This is a partial list. The full list is in Table S4.

b genetic position.

c marker type, P: Placement, not as accurate as Framework (F).

d Sequence Source, marker names with no GenBank accession number indicated that the sequences are available at http://www2.genome.arizona.edu/genomes/maize.

e positions in B73 RefGen_v1.

After integration, 97.8% of the physical map could be assigned to the maize genetic map, as compared to 86.1% [18] prior to the genome sequence. Among the 435 contigs in the updated physical map, 392 could be anchored, totaling 2073 Mb (97.8% of the 2120 Mb physical map). Among these 392 anchored FPC contigs, 163 (totaling ∼1222.9 Mb; 57.7% of the physical map) could be ordered and oriented in the maize genome, 92 (comprising ∼387.4 Mb) could be ordered, but not oriented, and 137 (∼462.8 Mb) had only rough genomic positions and were not ordered and oriented. Finally, the genomic positions of 43 FPC contigs (∼47 Mb; 2.2%) could not be determined due to lack of any sequence overlap and/or genetic linkage information. Development and mapping of polymorphic genetic markers from these latter contigs would be the most efficient approach to incorporate them into the integrated genetic and physical map of maize or other species.

### Ordering and orienting maize physical contigs using the maize optical map

**Figure 2:**
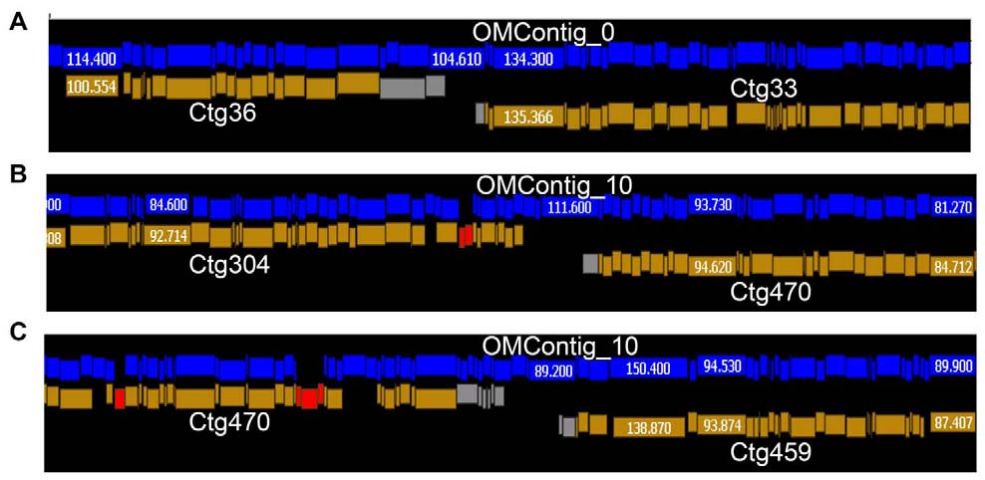
Use of the maize optical map for FPC contig anchoring. In each panel, the top blue fragments represent a maize optical *Swa*I restriction map, and the bottom orange fragments represent the *in silico* optical *Swa*I restriction map from contig-based pseudomolecules. Red fragments in (B) and (C) indicate a mis-sassmbly in the pseudomolecule that required manual editing. (A) Well-anchored Ctg36 helped to orient Ctg33, which was previously only ordered, but not oriented. (B) Anchored Ctg407 aided order and orientation of Ctg470, which was neither ordered nor oriented. (C). The newly anchored Ctg470 facilitated ordering and orienting of Ctg459.

Zhou et al. [19] reported the construction of an optical map for the B73 maize genome. The optical map was constructed by generating *Swa*I restriction maps of high molecular weight genomic DNA at 400-fold redundancy. The restriction maps were assembled into a whole genome optical map consisting of 66 contigs, many fewer than the 435 contigs in the maize physical map. To interdigitate the optical map with the integrated physical and genetic maps, we generated a contig-based *in silico* maize optical map by digesting the contig-based pseudomolecules (described below) with the *Swa*I restriction enzyme. The resulting *in silico* restriction map was then aligned to the maize optical map (see details in [19]) and used to assist with the ordering and anchoring of additional FPC contigs. For example, in Figure 2A, Ctg33 was well anchored on maize Chr1, while Ctg36 was only ordered but not oriented. Both FPC Ctg33 and 36 were mapped adjacent to one another in the maize optical map (Omcontig_0) thus allowing Ctg36 to be oriented correctly. In another example (Figure 2B and 2C), Ctg304 was well anchored (ordered and oriented) on maize Chr7, but the chromosomal positions of Ctg459 and 470 were unknown. These three contigs mapped next to each other in the following order: Ctg304, 470, and 459 on maize Omcontig_10. These data provided a genome context for the two orphan FPC contigs (Ctg459 and 470).

Combining the optical map analysis with the improved integrated genetic and physical map, we were able to anchor an additional 13 FPC contigs to the maize genetic map, which resulted in a final total of 405 anchored FPC contigs comprising 99.2% of the 2120 Mb physical map. More importantly, more than twice as many FPC contigs (336 as opposed to 163), comprising 94.0% of the physical map (∼1993 Mb), could be ordered and oriented. For the remaining contigs, 21 (containing ∼20.6 Mb) could be ordered, but not oriented; and 48 (∼90.1 Mb) had only approximate genomic positions and were neither ordered nor oriented. The final 17.1 Mb contained 30 contigs with no genome context. The efficiency of using the maize optical map for anchoring is remarkable due to its deep coverage, large single molecule, and contig sizes.

The anchoring quality of each contig, including the evidence used for anchoring, ordering and orienting, is shown in Table 2 (partial; the full list is in Table S5). The final integrated genetic and physical map can be downloaded at: http://www2.genome.arizona.edu/genomes/maize.

**Table 2:** Contig anchoring quality and contig positions in B73 RefGen_v1^a^.

Contig	Genetic Position	Order/Orien^b^	Number of Clones	Number of Markers	Physical Length (Kb)	Chr	Start^c^	End^c^
1	2.5	2	276	98	2523	1	1	2299274
2	13.5	2	132	53	1092	1	2300275	3419854
3	26.1	2	334	160	2126	1	3420855	5929995
4	82.8	2	616	262	4165	1	5930996	10045647
5	103	2	350	152	3224	1	10046648	13079531
6	124.7	2	459	172	2851	1	13080532	16193432
7	145	1	109	44	1156	1	16194433	17299506
8	160.6	2	793	280	5600	1	17300507	23505871
9	170	2	801	249	5933	1	23506872	29869500
10	205	2	2427	745	18541	1	29870501	48303993
12	290.1	2	506	179	4277	1	48304994	52419976
13	292.4	2	72	35	715	1	52420977	53153490
14	325.7	2	1672	434	12700	1	53154491	65772465
16	360.9	3	281	77	1920	1	65773466	67717626
474	385	0	79	39	705	1	67718627	68401065
17	386.4	2	247	63	2361	1	68402066	70727174
18	391.8	3	379	119	3189	1	70728175	73586599
19	392.95	3	626	136	4640	1	73587600	78384554
20	398.2	2	798	198	6350	1	78385555	84665058
22	406	3	253	68	1832	1	84666059	86598526
24	415	3	482	99	3973	1	86599527	90264916
23	417	2	578	114	4395	1	90265917	94733311
106	227.1	4	939	190	7359	9	53598148	60378895
432	227.2	4	701	135	4875	9	60379896	65011726
448	227.3	4	576	90	4512	9	65012727	69221205
425	unknown	5	218	51	1871	0	9718511	11319527
427	unknown	5	236	35	2033	0	11479785	13269771
429	unknown	5	166	41	1759	0	13443808	14680007

a This is a partial list. The full list is in Table S5.

b Code: 0, chromosomal assignment is known, but not ordered and oriented; 1, ordered, but not oriented; 2, genetically anchored and oriented; 3, anchored and oriented with assistance from optical map; 4, the block was anchored, but order and orientation are unknown; 5, unknown chromosomal context.

c positions in B73 RefGen_v1.

### Generation of A Golden Path (AGP) of the maize B73 genome

A major objective of the MGSC was to sequence the genome, integrate the sequence into the maize genetic and physical maps, and provide a high quality reference sequence in low copy regions. The final step of the MGSC, before annotation, was to generate a set of ten pseudomolecules that represented the ten chromosomes of maize—called “a golden path” or “AGP.” AGPs greatly simplify the analysis of a genome because an AGP removes all redundant overlapping sequences between BACs and fosmids, and provides a convenient set of contiguous sequence for annotation, as opposed to having to download over 16,000 individual BAC sequences and assembling them into a genome sequence independently. Most BAC sequences, generated by the MGSC and deposited in GenBank, contained multiple sequence contigs (on average 11 per clone) some of which were neither ordered nor oriented; it was thus very challenging to construct the AGP.

**Figure 3:**
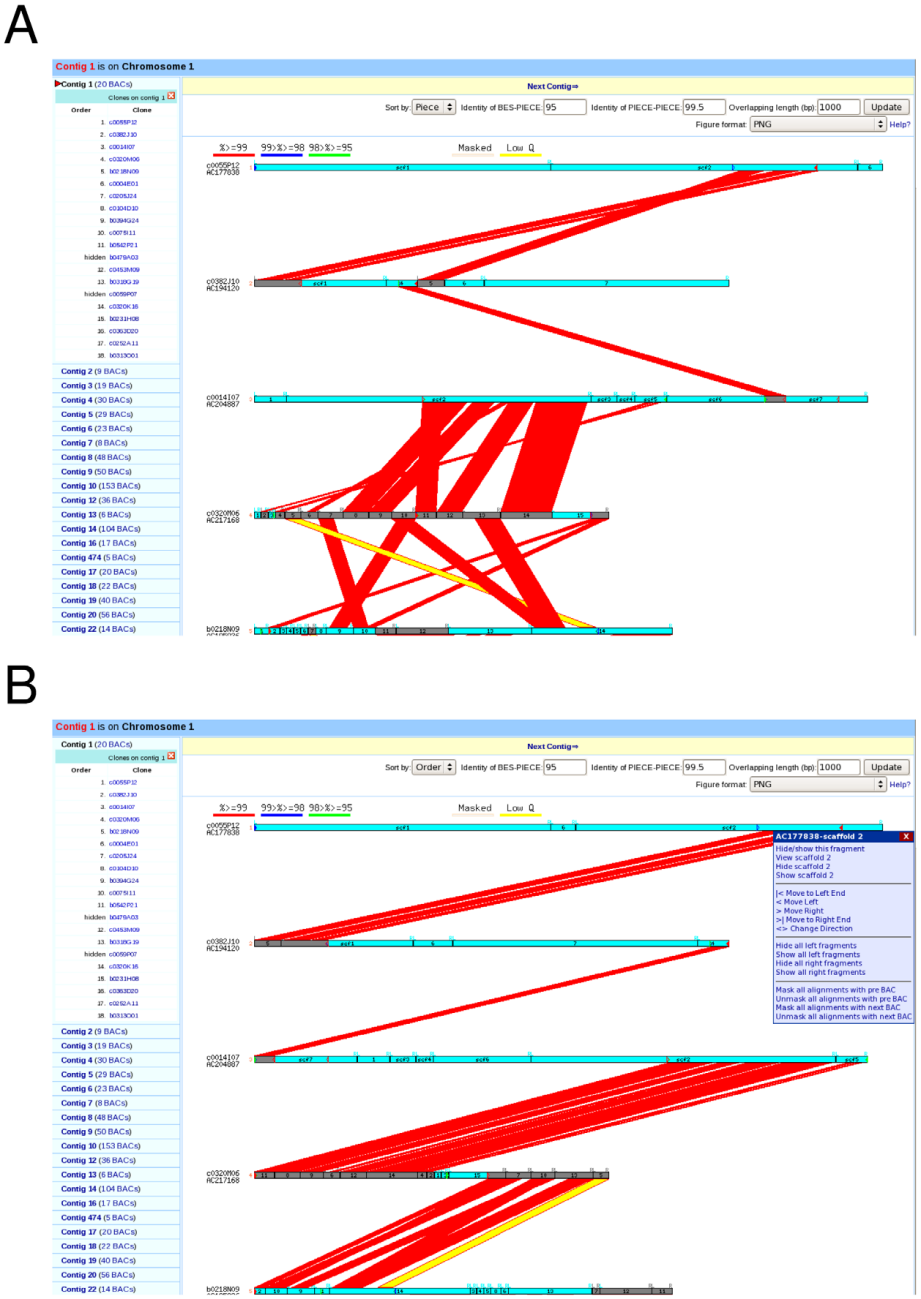
Direct comparison of sequence overlap between adjacent clones before (A) and after (B) the semi automated AGP pipeline.

The main task in building a whole genome AGP is to determine the extent of overlapping sequence between adjacent clones, order and orient sequence contigs in the overlapping regions, and finally remove all redundant overlapping sequence. To accomplish this task, we built a semi-automated web-based AGP pipeline connected to a MySQL relational database that was run with custom Perl scripts. All available sequence data including BAC, BAC and fosmid –end, and marker sequence information from both the MGSC and outside projects were then loaded into the MySQL database. A set of comparisons was then performed between neighboring BAC sequences and/or BES using BLAST, which resulted in the identification of the left and/or right end of each BAC on adjacent BACs, as well as overlapping sequence between two adjoining clones. Employing a user-friendly graphical interface (Figure 3A and 3B), we manually curated the order and orientation of BAC pieces in overlapping regions, and removed overlapping or redundant sequences from the final pseudomolecule according to sequence alignment. All processing information was saved into our database for creating the AGP file.

At present, a total of 16,910 clones assigned to 435 FPC contigs have been processed by the AGP pipeline. After removal of sequence overlap and ordering and orienting sequence contigs within the overlapping regions, we were able to generate a maize AGP composed of 2048 Mb of pseudomolecule sequences in 61,161 scaffolds from 125,325 sequence contigs, which covers ∼97% of the 2120-Mb physical map. Table 3 summarizes the sizes, scaffolds, and contig number of each maize chromosome plus those that are unanchored. The AGP and maize B73 RefGen_v1 are available at: http://www2.genome.arizona.edu/genomes/maize.

**Table 3:** Sequence summary of the maize chromosomes in B73 RefGen_v1.

Chr	Length (bp)	Scaffold			Contig		
		Number	Length (bp)	Average (bp)	Number	Length (bp)	Average (bp)
**0** a	14680007	647	14588907	22549	1206	14531607	12049
**1**	300239041	8696	299312341	34420	17683	298405441	16875
**2**	234752839	6661	234044439	35137	13694	233333939	17039
**3**	230558137	6612	229865037	34765	13509	229167937	16964
**4**	247095508	6834	246365608	36050	13975	245638708	17577
**5**	216915529	6547	216219929	33026	13148	215551729	16394
**6**	169254300	5257	168698300	32090	10986	168119000	15303
**7**	170974187	5239	170418187	32529	10858	169851287	15643
**8**	174515299	5452	173935699	31903	11450	173330599	15138
**9**	152350485	4653	151852185	32635	9243	151386685	16379
**10**	149686045	4563	149202145	32698	9573	148697745	15533
**Total**	**2061021377**	**61161**	**2054502777**	**33592**	**125325**	**2048014677**	**16342**

a total of all unanchored contigs.

### Conclusion

We used an integrated genetic and physical map to select and validate an MTP of clones across the maize genome as the template to generate a whole genome sequence. Using individual BAC assemblies, over 8,300 sequence-based genetic markers, and the optical map, we significantly improved the integrated genetic and physical map of maize, which in turn resulted in the generation of an AGP across the maize genome. The tremendous resources generated by this project will greatly facilitate basic and applied research on multiple fronts, including comparative and functional genomics studies, genome structure and evolution, map-based gene cloning, and molecular breeding.

Although the first release of the maize genome (i.e. B73 RefGen_v1) is now realized, as with any genome sequence, several improvements are still needed to produce an even more accurate reference sequence for maize. First, six percent of the genome (127.8 Mb in total) still needs to be genetically ordered and oriented. This includes 20.6 Mb (1.0% of the physical map in 21 contigs) to be oriented, 90.1 Mb (4.3% in 48 contigs) to be precisely ordered and oriented, and finally, 17.1 Mb (0.8% in 35 contigs) to be genetically mapped. Secondly, the physical map covers ∼93% of the B73 genome in 435 contigs, and significant physical gaps remain to be bridged. For example, approximately 5% of the maize full-length cDNA data set could not be mapped to the genome (i.e. B73 RefGen_v1; [26]). Finally, we must continue to better orient sequence contigs within BACs using multiple data types, such as the optical map, syntenic relationships across the cereal genomes, full-length cDNA evidence, and paired-end whole genome shotgun sequence. Data generated from the maize diversity project should provide enough evidence to anchor most unanchored contigs (Ed Buckler, pers. comm.). Efforts to further improve the B73 RefGen_v1 are now underway, and new AGP releases will be made available regularly through the AGI website (www2.genome.arizona.edu/genome/maize).

## Materials and Methods

### Physical map editing and anchoring

All steps related to physical map editing were as previously described [18].

### Sequence based genetic marker integration

See Text S1.

### MTP clone selection pipeline

See Text S1.

### AGP generation pipeline

See Text S1.

## Supporting Information

Table S1 MTP clones and their physical position, sequence characteristics, and overlap information.
(2.82 MB XLS)

Table S2 Contig number and orientation change after merging and breaking.
(0.03 MB XLS)

Table S3 Genetic markers and their genetic, physical, and RefGen_v1 positions.
(1.80 MB XLS)

Table S4 The position of bin markers in the B73 physical map and RefGen_v1.
(0.03 MB XLS)

Table S5 Contig anchoring quality and contig positions in B73 RefGen_v1.
(0.05 MB XLS)

Text S1 The maize MTP pipeline, the maize AGP pipeline, and sequence-based genetic markers.
(0.40 MB DOC)
