# Supplementary material for: The Physical and Genetic Framework of the Maize B73 Genome
Source: PLoS Genet. 2009 Nov 20;5(11):e1000715. doi: 10.1371/journal.pgen.1000715 (PMC2774505; doi:10.1371/journal.pgen.1000715)
Supplement: Text S1 — The maize MTP pipeline, the maize AGP pipeline, and sequence-based genetic markers. (0.40 MB DOC) [file pgen.1000715.s006.doc]

**The Maize MTP Pipeline**


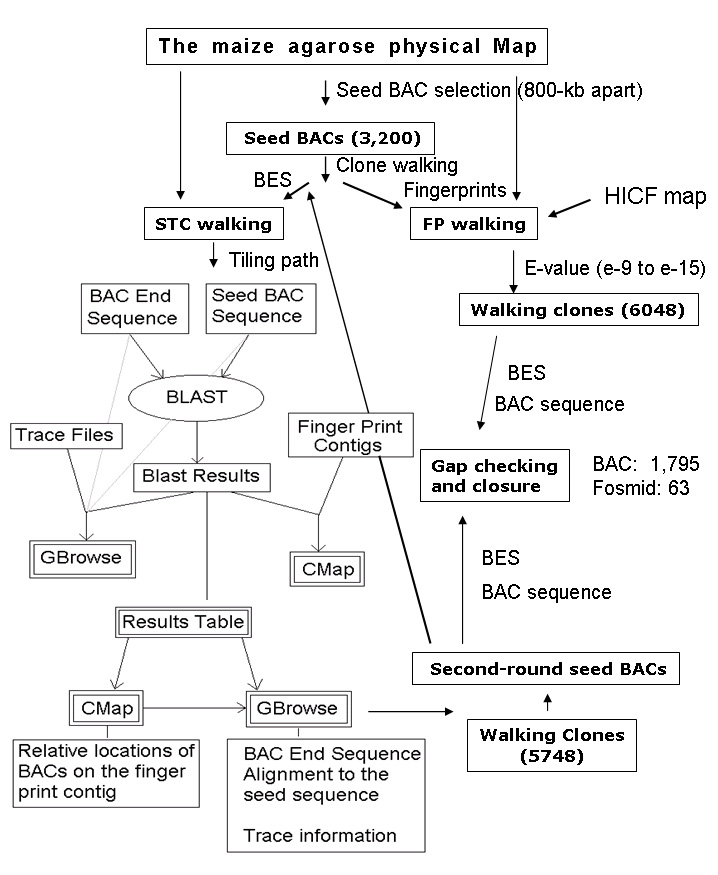


Flowchart for minimum tiling path BAC clone selection across the maize genome.

Initially 3,200 seed BACs (~ one clone per 800 kb on physical map) were picked. The following criteria were applied to seed BAC selection:

1) Genomic inserts were larger than average of the BAC libraries.

2) High-quality paired-end sequence data was available.

3) High-quality fingerprint data was available.

4) A genetic and/or overgo marker was associated with each seed BAC where possible.

After seed BAC clones were sequenced, assembled and improved, two approaches were adopted for selecting additional MTP BAC clones, STC (sequence tagged connector)-based walking, fingerprint-based walking.

A) STC-based clone walking:

1. Retrieve BAC/Fosmid end sequences for STC walks from “Trace Archives” at NCBI (<http://www.ncbi.nlm.nih.gov/Traces/home/>). Two queries were used to obtain these end data from the trace archive:
2. *CENTER_NAME='WUGSC' and SPECIES_CODE='Zea mays' and TRACE_TYPE_CODE= 'cloneend' and LIBRARY_ID='CH201'*
3. *CENTER_NAME= 'WUGSC' and SPECIES_CODE='Zea mays' and TRACE_TYPE_CODE='cloneend' and LIBRARY_ID= 'CH20'*
4. *LIBRARY_ID='Z_AI'* for 827,571 for fosmid-end trace files
5. A web-based MTP Tilepath pipeline interface for the sequenced tagged connector approach (STC walks) was developed by utilizing the BES data set to identify BESs that minimally aligned to seed BAC (and subsequent BAC-walk) sequences on either side of the sequence.

In the MTP Tilepath pipeline:

1. Assembled sequences from seed BACs were searched against the maize BESs database.
2. Results were classified based on location on the physical map.
3. A table for each BAC was created of filtered BLAST results with links to CMap and GBrowse.
4. BLAST results were imported into CMap and GBrowse with additional information such as trace files and FPCs.
5. A table of alignments between the seed BAC (and subsequent BAC-walks) and BAC end sequences contained links to CMap and GBrowse.
6. CMap displayed the FPC data for seed BACs (and subsequent BAC-walk) and the candidate BAC clones to pick.
7. GBrowse provided an alignment of BESs with seed BAC sequences (and subsequent BAC-walk sequences) and displayed the trace file data.
8. The procedure to use the MTP Tilepath pipeline is shown in Figure S1.
9. When the clone name of a seed BAC was selected from the list, a new interface popped up to show a list of potential walking clones along with sequence identities.
10. When crosschecking the list with the maize agarose FPC map, a candidate walking BAC could be identified.
11. The candidate BAC clone was subjected to further analyzsis by clicking Gbrowse next to the clone trace name (Fig. S2A).
12. The Gbrowse interfaces allowed us to visualize the alignment of seed BAC sequences and BESs of a candidate BAC clones along with BES trace chromatograms (Fig.S2B).
13. This process ensured the sequence alignment was as accurate as possible. It should be noted, that we used trace file data because it is well known that single-pass BESs are prone to higher levels of sequence errors primarily due to technical issues involving “BigDye blobs” during BAC end sequencing. Sequence identity, such as 98% between a seed-BAC sequence and BESs of its neighbors did not give us enough confidence to guarantee a precise overlap. We therefore carefully inspected and confirmed all sequence alignments using trace chromographs before a new walking BAC clones would be selected.

B) Fingerprint-based clone walking:

We used the maize HICF physical map for BAC clone walking for the following reasons. First, fingerprints generated using the HICF method yielded, on average, 115 bands vs. 30 using the agarose method. Therefore it was easier to identify clones with minimum overlap due the high resolution of the HICF fingerprints. Secondly, both the agarose and HICF maps were constructed using the same set of clones, but different fingerprinting technologies. Even though the HICF map was not manually edited, we could refer to the agarose map for contig validation. To ensure a true but minimum overlap, a MTP clone was selected in the HICF map only if the shared e-value with its neighboring clones was between e^-9^ and e^-15^. E-values were assessed using the FPC Analysis function.

C) After a BAC or fosmid clone was selected from the above pipeline, the clone was validated by re-end sequencing, both before preparation for shotgun library construction and after DNA shearing, prior to end-polishing, ligation and *E. coli* transformation.

D) To check gaps between MTP clones, we developed another web-based MTP interface to ensure true overlap between two BAC clones. The gap checking/filling criteria were set as following:

1) Two adjoining clones must have a 99.9% sequence overlap (or greater) using Megablast and trace file data.

2) The highest scoring overlap region must only belong to adjoining clones, and not to any other clone(s) mapped to other genomic regions.

3) The BES of one clone must align to the sequence of its adjacent clone with over 95% identity;

4) If the sequence identity in the BAC-end search was less than 99%, the sequence alignment along with the trace chromatograph was manually checked.

If any one of these criteria were not met, a gap would be flagged and manually checked. A confirmed gap would be filled with either a BAC or fosmid clones by STC walking.

**Maize AGP Pipeline**

The outline of the maize AGP generation is shown in Fig.3A. The whole process can be described as follows.

1. Collect all BAC, BAC and fosmid-end, and genetic marker sequences.
2. Import MTP, BESs, FESs, genetic markers and marker sequences into a MySQL database.
3. Split BAC sequences into pieces by gaps, but keep scaffolds, then load into a MySQL database
4. Run batch BLAST searches without filter (-F F): BESs against BAC pieces (bl2seq -e 1e-20), sequence pieces of two adjacent BACs against each other (bl2seq -e 1e-200), marker sequences against BACs (BLASTn -e 1).
5. Initially order BAC pieces and remark overlap regions according to alignment among pieces of two adjacent BACs (identity >= 99.5%, match length>= 1 kb).
6. Find left and/or right end of each BAC, determine and re-mark orientation (first level) of related pieces by checking BLAST hits between BESs and BAC pieces (identity >= 95%).
7. Reorder BAC pieces and adjust piece orientations according to the first-level oriented pieces (BAC end related) and the relationships among pieces in overlap regions.
8. Manually check (and adjust if needed) the order and orientation of BAC pieces in overlap regions, and mask the overlap or redundant sequences clone-by-clone with a user friendly web-based graphical interface. Some regions with identities of less than 99.5%, or matches shorter than 1 kb, also were marked as overlap or redundant when considering the possibility of sequence errors or low coverage.
9. Manually execute specific operations (a, b, c) from a pop up menu for different purposes:

a) to hide or show a whole redundant clone and run BLAST comparisons between related clones;

b) to mask overlap regions to keep one copy of a sequence for the final output;

c) to individually adjust order or to orient a contig or scaffold within a BAC.

1. Create FPC-contig-based pseudomolecules.
2. Generate an AGP based on improved physical map that was integrated with the genetic map by referring to BLAST results of marker sequences against BACs.

**Sequence-based Genetic Markers**


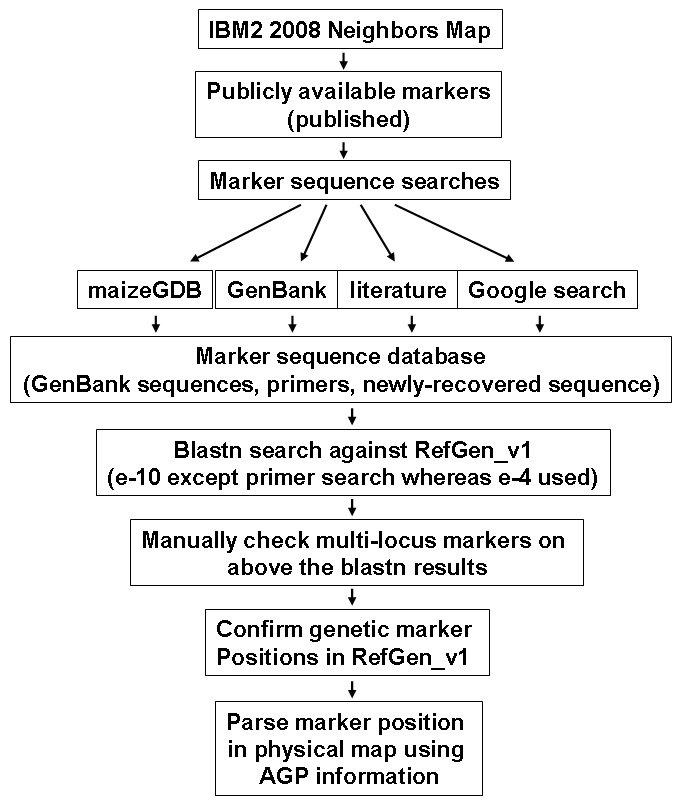


Flowchart of integration of genetic markers into the maize physical map and B73 RefGen_v1.

To integrate the maize genetic map with physical map and the AGP at a high resolution we used *in silico* hybridization with publicly available sequence-based genetically mapped markers. The outline for the integration of genetic markers into the physical map and RefGen_v1 is as follows:

- 1. **Marker Collection.** Due to the long history of many genetic markers, not all sequences of these markers have been deposited in the public domain, such as GeneBank or maizeGDB. Integration of genetic markers to the physical map and RefGen_v1 is valuable for the research community and for anchoring physical contigs to the maize genome. To collect the sequences of as many published genetic markers as possible, we downloaded markers from: maizeGDB ([www.maizeGDB.org](http://www.maizeGDB.org/)), Genbank (www.ncbi.nlm.nih.gov), the literature, and Google searches

For the marker sequences not deposited in GenBank or maizeGDB, because we did not generate the sequences, we cannot deposit them into any central public database. However, all sequences collected are available at the AGI website <http://www2.genome.arizona.edu/genomes/maize>.

2. **Genome Searches**. BLASTn was used to find marker sequences in the physical map and the pseudomolecules. Except for primers, e-values less than e^-10^ were used in BLASTn searches There were no clear cutofffs in terms of e-values to parse the BLASTn results. For multiple loci markers with only one sequence, various e-values were applied to find the best positions for these markers. For primers, we used an e-value less than e^-4^. Here we noticed some inconsistences of genetic positions of primer mapping and their original sequence mapping due to primer mismatch in PCR amplification.

3. **Manual Checking**. After we obtained the BLASTn results, the data were parsed according to their genetic positions and then manually checked for accuracy.

4. **Genome Position**. Once every data point was confirmed, we then integrated them with the physical map. This integration allowed us to anchor and orient additional FPC contigs to the genetic map, which in turn were used to dynamically correct the AGP (RefGen_v1).
